# Integrated Analysis of the Immune Infiltration Pattern and Novel Diagnostic Biomarkers in Septic Cardiomyopathy

**DOI:** 10.1002/iid3.70311

**Published:** 2026-01-19

**Authors:** Wei Liu, Xi Zheng, Dong Wang, Jingyi Wang, Xincheng Li, Fei Li, Wenxiong Li, Jin Zhang

**Affiliations:** ^1^ Department of SICU, Beijing Chaoyang Hospital Capital Medical University Beijing China; ^2^ Department of Orthopedics, Beijing Chaoyang Hospital Capital Medical University Beijing China

**Keywords:** hub genes, immune infiltration, septic cardiomyopathy, WGCNA

## Abstract

**Purpose:**

In this study, various informatics analyses were employed to identify the hub genes associated with septic cardiomyopathy (SCM) onset and investigate their immune infiltration status.

**Methods:**

High‐throughput sequencing data of myocardial tissue samples from mice with SCM were obtained from the GEO database and our previously published articles. The Limma and weighted gene co‐expression network analysis (WGCNA) packages were used to identify the hub genes associated with SCM onset. GSEA and the DAVID database were employed for gene enrichment analysis. Additionally, the CIBERSORT database was used to analyze the immune infiltration in SCM. Finally, the multiMiR package was used to analyze the microRNAs acting as ceRNAs for the hub genes. Receiver operating characteristic (ROC) curves and Mendelian randomization analysis were used to evaluate the predictive value of hub genes for SCM.

**Results:**

The SCM group included nine samples, while the control group included ten samples. SCM upregulated 15 genes and downregulated 7. *Mt1* and *Actc1* were the most significantly upregulated and downregulated, respectively. GO analysis indicated that the most significantly enriched biological process was “response to bacterium,” and the most enriched signaling pathway was “mineral absorption.” Immunoinfiltration analysis revealed decreased T cells CD4 naive, B cells naive, resting mast cells, and M2 macrophage infiltration in the hearts of SCM mice. WGCNA and Limma package analyses identified *Clu*, *Igf1*, and *Trp53* as hub genes associated with SCM onset. The ROC curves demonstrated a strong correlation and predictive value for *Trp53*, *Igf1*, and *Clu* in the SCM. Moreover, *Clu and Igf1* demonstrated predictive values for SCM using Mendelian randomization analysis from the IEU database. Eleven miRNAs formed a ceRNA network with these hub genes.

**Conclusion:**

In summary, our results implicated *Igf1* and *Clu* as the potential candidates involved in SCM pathogenesis.

## Graphical abstract

In this study, various informatics analyses were employed to identify hub genes associated with the onset of septic cardiomyopathy and investigate its immune infiltration status.

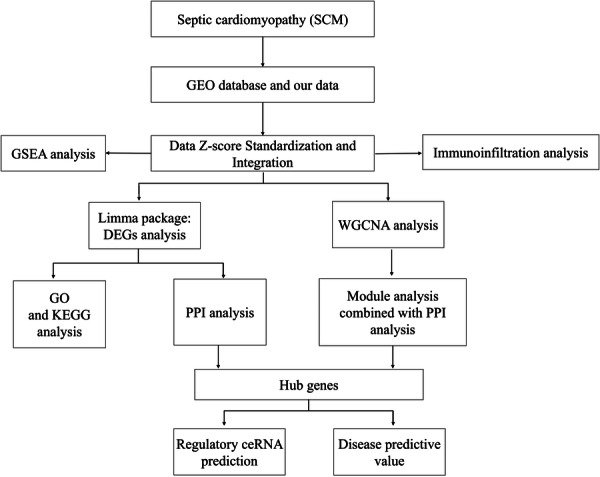

**Abbreviations**
BP: biological processes
CC: Cellular Components
CLP: cecum ligation and puncture
DEGs: differentially expressed genes
GEO: Gene Expression Omnibus
GO: Gene Ontology
GSEA: Gene Set Enrichment Analysis
KEGG: Kyoto Encyclopedia of Genes and Genomes
LPS: lipopolysaccharide
MF: Molecular Functions
PCA: principal component analysis
PPI: Protein‐protein Interaction
ROC: receiver operating characteristic
SCM: septic cardiomyopathy
WGCNA: weighted gene co‐expression network analysis

## 1 Introduction

Septic cardiomyopathy (SCM) is a serious cardiac dysfunction that occurs during sepsis, particularly during septic shock. Current clinical observation study found SCM was a reversible process if sepsis cured [1, 2]. Clinically, SCM‐induced cardiogenic shock and SCM‐distributed shock are often mixed together [3]. Cardiogenic shock progresses extremely fast after it onsets, demanding usually invasive cardiac support. Some patients even lose the window for intervention and are left with an unfavorable prognosis [4]. It has become a major focus of current research.

SCM, also known as sepsis‐induced cardiomyopathy, is a secondary cardiac insufficiency caused by sepsis, and its components are related to an exaggerated immune response. Various pathways, such as adrenergic pathway dysregulation, energetic failure due to mitochondrial dysfunction, downregulation of sarcomeres, and abnormalities in calcium handling, may be involved in SCM [1, 3]. However, the development of high‐speed sequencing technologies has not only focused on the molecular and cellular levels but also on gene expression difference [5, 6, 7].

Some databases established to analyze the key genes and pathways of SCM se different intervening measures, experimental animal species, and sampling times. Presently, the main SCM model in animals is the cecum ligation and puncture (CLP) model, but the cardiac function index is very unstable [5, 6]. The lipopolysaccharide (LPS)‐injected model is controversial because of the poor systemic inflammatory response and high dose [8, 9, 10]. Our team built an SCM model by *Pseudomonas aeruginosa* incision infection [7]. Each model presented different gene expression profiles and identified various pathways related to pathophysiological processes. Analysis of the common gene pathways in different intervening measures may enhance our understanding of the molecular basis.

This study continuously explored the mechanism of action of SCM using different intervention measures in mouse models. Our database and the GSE171546 and GSE229925 databases published by Zhang et al. and Yan et al., respectively, were reanalyzed [5, 6]. We analyzed the sequencing data derived from cardiac tissues 24 h after SCM induction. In Series GSE171546, cardiac mRNA profiles were retained for 24, 48, and 72 h, we chose the 24 h profiles [5]. In Series GSE229925, CLP‐induced mice were divided into three groups based on left ventricular ejection fraction (LVEF), and the low LVEF group (LEF, LVEF < 65%) was chosen as the experimental group [6].

*Il‐6*, *Il‐1β*, and *Tnf* were the hub genes of SCM according to analyze our database, which encode cytokines mostly derived from immune cells. Our previous study attempted to build a model of myocardial apoptosis in vitro in a septic microenvironment. We found that LPS could not be used to establish a stable apoptotic model in myocardial cells. This prediction was confirmed using the macrophage co‐culture model. Based on the above experimental results, we speculate that immune cells are required for the SCM process. Immune infiltration analysis was used to clarify immune cell distribution and function in the SCM. We used the “Limma” package and weighted gene co‐expression network analysis (WGCNA) to obtain the differentially expressed genes (DEGs), built the key ceRNA networks, and used to predict the occurrence of SCM. This provides a basis for exploring the pathophysiological processes and pharmacological interventions. Mendelian randomization analysis was used to further verify the correlation between the hub genes and SCM.

## 2 Material and Methods

### 2.1 Data Acquisition

**Figure 1:**
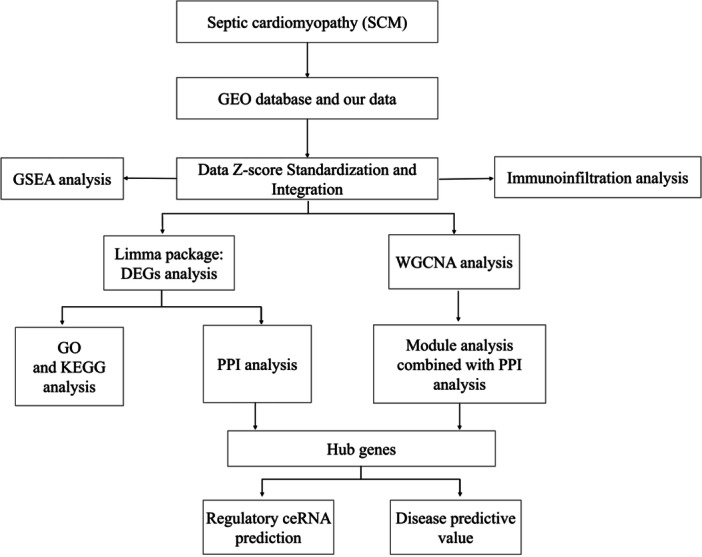
Flowchart of integrated analysis to identify key genes associated with septic cardiomyopathy.

In this study, microarray data were obtained from the Gene Expression Omnibus (GEO) database (https://www.ncbi.nlm.nih.gov/) and from article authored by our research team [7]. The GEO database was queried using the following keywords: “septic cardiomyopathy”, “sepsis‐induced cardiomyopathy”, or “septic shock” with the species specified as mice. We opted to analyze the sequencing data derived from cardiac tissues 24 h after the induction of septic cardiomyopathy. Data were excluded if the sampling time was not clearly specified, if the modeling methods were unclear, or if the data did not pertain to in vivo cardiac tissue sequencing results. The analytical process of this study is illustrated in Figure 1.

### 2.2 Data Integration and Differentially Expressed Genes (DEGs) Analysis

Initially, individual datasets underwent meticulous processing. Sequencing results for each sample were extracted from the datasets, with gene names represented using gene symbols. For duplicated genes, calculated the mean value for representation. Subsequently, the sequencing data underwent Z‐score standardization. The standardized datasets were then integrated, resulting in a total of nine samples from the septic cardiomyopathy (SCM) group and 10 samples from the control (C) group.

The “limma” package was employed in R software for the analysis of DEGs within the integrated dataset [11]. DEGs were defined based on criteria, such as a p‐value less than 0.05 and a log2FC (log2 fold change) value greater than 1 or less than −1. The DEGs were subjected to principal component analysis (PCA) using the origin 2021 platform. This rigorous analytical approach aimed to identify and characterize significant molecular variations associated with septic cardiomyopathy, providing a foundation for deeper insights into the underlying genetic mechanisms.

### 2.3 Functional Enrichment Analysis of DEGs and Protein‐Protein Interaction (PPI) Analysis

The DAVID database (https://david.ncifcrf.gov/home.jsp) was employed for the Gene Ontology (GO) and Kyoto Encyclopedia of Genes and Genomes (KEGG) analyses of DEGs [12, 13]. GO analysis comprised Biological Processes (BP), Cellular Components (CC), and Molecular Functions (MF). Enrichment was considered significant when the *p*‐value was less than 0.05.

The STRING 12.0 database (https://string-db.org/) was utilized to construct a protein‐protein interaction (PPI) network for DEGs, concurrently excluding isolated proteins [14]. Following network construction, Cytoscape 3.10.1 software and cytoNCA app facilitated the analysis of PPI results, involving the computation of various network metrics such as betweenness, closeness, and degree for each protein [15, 16, 17]. Three algorithms were applied to rank genes based on their betweenness, closeness, and degree within the PPI network. The top‐ranking gene from each algorithm was selected as a hub gene.

### 2.4 Weighted Gene Co‐Expression Network Analysis (WGCNA)

In the R software, the “WGCNA” and “tidyverse” package were employed for the comprehensive analysis of gene co‐expression networks. The analysis was conducted on a dataset that had been integrated after Z‐score standardization. The selected phenotype for this analysis was septic cardiomyopathy.

The WGCNA analysis involves the construction of a co‐expression network based on the integrated and standardized Z‐score dataset. Modules, representing clusters of co‐expressed genes, are identified through hierarchical clustering. Subsequently, the correlation between these modules and the chosen phenotype (SCM) was assessed. Modules exhibiting significant correlations with the phenotype were selected for further analysis, with a threshold set at a p‐value less than 0.05.

Similarly, the STRING database was employed to construct PPI networks for modules exhibiting correlation [14]. Cytoscape software, in conjunction with the cytoNCAapp, was utilized for the computation of various parameters within the PPI networks [15, 16, 17]. Key network metrics, including betweenness, closeness, and degree, were calculated for each gene. Genes ranking first in betweenness, closeness, and degree within the computed network parameters were selected as hub genes.

### 2.5 Gene Set Enrichment Analysis (GSEA)

The GSEA analysis was conducted utilizing several specialized R packages, including “clusterProfiler”, “ReactomePA”, “org. Mm. eg. db”, “tidyverse”, “biomaRt”, and “nrichplot”. The significance threshold for enrichment is set at a *p*‐value of 0.05.

### 2.6 Immune Infiltration Analysis

The CIBERSORT database (https://cibersort.stanford.edu/) was employed for the comprehensive analysis of immune infiltration [18]. Significance analysis involved setting permutations to 1000, utilizing a selection of 22 immune cell datasets. The data results were visualized using Origin 2021 and GraphPad Prism 8.

### 2.7 Prediction of microRNA Regulation on Hub Genes

The R package “multiMiR” was utilized for the analysis of microRNAs capable of regulating hub genes. Following the prediction phase, the “ggvenn” package was employed to construct Venn diagrams.

### 2.8 Analysis of Disease Prediction Impact

The software MedCalc version 20.014 was employed for analyzing the strength of hub gene predictions in septic cardiomyopathy. The DeLong test was utilized to compare the differences among receiver operating characteristic (ROC) curves. The above databases were used to build the ROC curves. SCM as the dependent variables, while the expression of hub genes as the independent variables.

### 2.9 Mendelian Randomization Analysis

The IEU database (https://opengwas.io/datasets/) was used to obtain the single nucleotide polymorphisms (SNPs) data of septic cardiomyopathy and hub genes. The devtools, TwoSampleMR, VariantAnnotation and gwasglue packages in R language were used for data processing. To ensure the significance and correlation of the SNPs, we set the SNPs *p*‐value < 1e‐03. The harmonise_data function was used to integrate the exposure and outcome data to ensure data consistency and comparability. Meanwhile, we conducted heterogeneity analysis, and the mr_heterogeneity function was used to detect the variability among different SNPs. The mr_presso function was used for pleipotency testing, with NbDistribution set to 3000 to verify the validity of instrumental variables.

### 2.10 Statistical Analysis

Origin 2021 was employed for conducting statistical analyses in this study. Count data were expressed as percentages, and inter‐group comparisons were conducted using either the Chi‐square test or Fisher's exact test. For continuous data, mean values ± standard deviation were utilized. In cases where the data followed a normal distribution, inter‐group comparisons were performed using independent samples *t*‐test. Conversely, for non‐normally distributed data, inter‐group comparisons were conducted using the Mann‐Whitney *U* test. Statistical significance is determined by a *p*‐value less than 0.05.

## 3 Results

### 3.1 Data Selection and Grouping

**Figure 2:**
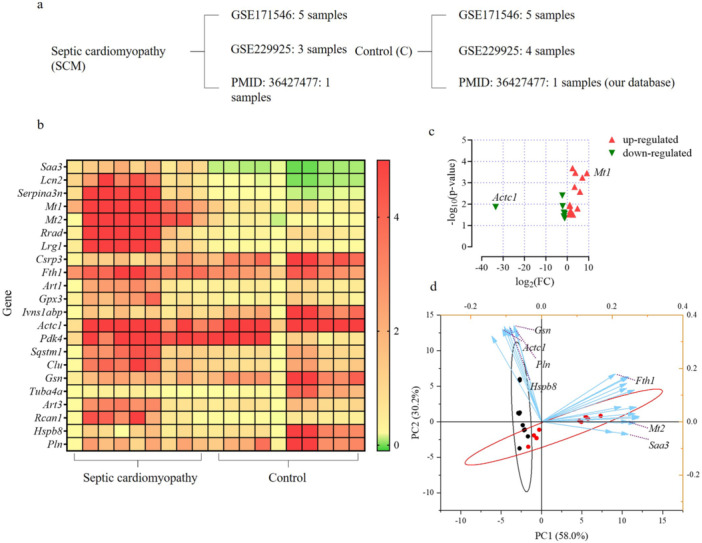
The datasets of the two groups and the differentially expressed genes. (a) the included datasets in the SCM group and the control group. (b) heat map of differentially expressed genes. The map showed 22 differentially expressed genes, among which 15 were up‐regulated and 7 were down‐regulated. (c) scatter plot. Mt1 was the most significantly upregulated gene and Actc1 was the most significantly downregulated gene. (d) PCA results. The red circles and the black circles represented the SCM group and the control group, respectively. The blue arrows represented the gene expression data.

Data were obtained from the GEO database and our research as well as the distribution of samples in the two groups. The SCM group included nine samples from GSE171546 (5 samples), GSE229925 (3 samples), and article PMID: 36427477 (1 sample). The control group included 10 samples from datasets GSE171546 (5 samples), GSE229925 (4 samples), and article PMID:36427477(1 sample; Figure 2a).

### 3.2 DEGs

After meticulous data selection, the final datasets incorporated into the study comprised GSE171546, GSE229925, and data from our published article [7]. Through Z‐score standardization and dataset integration, the SCM group ultimately comprised nine samples, while the control group consisted of 10 samples.

DEG analysis revealed that 22 genes exhibited significant differences in expression. In comparison to that in the control group, 15 and 7 genes were upregulated and downregulated in the SCM group, respectively (Figure 2). Notably, *Mt1* was the most significantly upregulated, while *Actc1* was the most substantially downregulated (Figure 2).

Principal component analysis (PCA) further elucidated the distinct gene expression patterns. In the septic cardiomyopathy group, the principal component genes were identified as *Fth1, Mt2*, and *Saa3*, whereas in the control group, *Actc1, Gsn, Hspb8*, and *Pln* were the principal component genes (Figure 2).

### 3.3 Gene Ontology and Kyoto Encyclopedia of Genes and Genomes Enrichment Analysis

**Figure 3:**
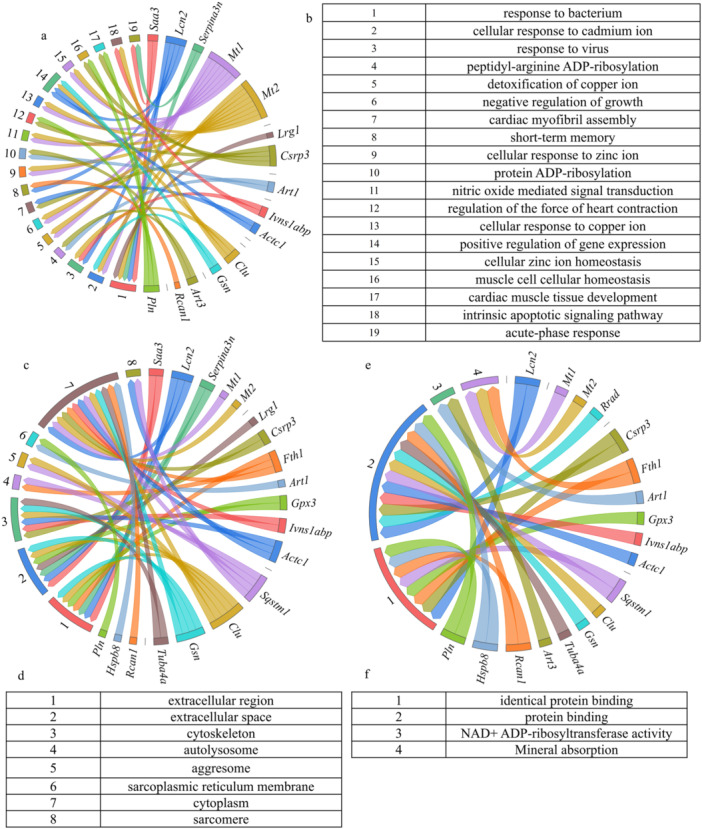
GO and KEGG enrichment analysis results. (a, b) BP results chord diagram. The figure showed the connection between BP terms and the corresponding genes. The table presented the names of BP terms. Among them, response to bacterium, cellular response to cadmium ion, and response to virus were the most important BP terms. (c, d) enrichment results of CC terms. The figure showed that cytoplasm, extracellular region, and extracellular space were the top three terms with the most recruited genes. (e, f) MF and KEGG enrichment results. Protein binding was the top term.

Gene ontology (GO) enrichment analysis highlighted notable associations of BP, CC, and MF with DEGs. The most significant BP identified was “response to bacterium,” which involved five genes. Subsequently, other prominent BP included “cellular response to cadmium ion” and “cellular response to cadmium ion.” In terms of CC, “extracellular region” emerged as the most significant, featuring seven participating genes, followed by “extracellular space” and “cytoskeleton.” The most significant MF was “identical protein binding,” succeeded by “protein binding,” (Figure 3).

Kyoto encyclopedia of genes and genomes (KEGG) enrichment analysis revealed prominent pathways associated with the DEGs. “Mineral absorption” was the most significant pathway, (Figure 3).

### 3.4 Protein–Protein Interaction Analysis

**Figure 4:**
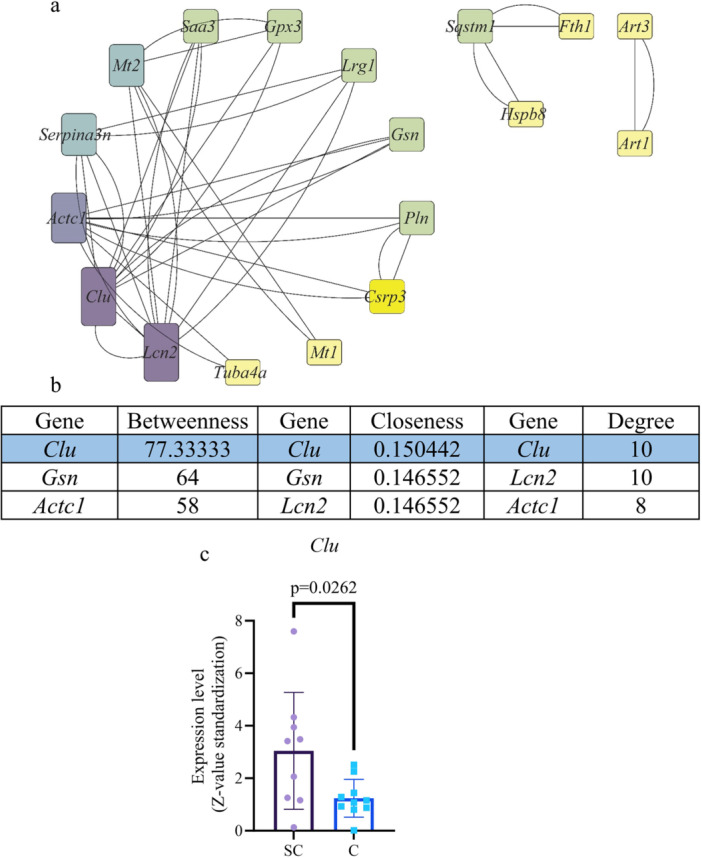
PPI network and hub gene analysis. (a) PPI network graph. The color scale represented the node degree. (b) PPI network analysis results. Three distinct algorithms were applied to analyze the PPI network, and Clusterin (CLU) was identified as the most important protein. (c) Expression of Clu in septic cardiomyopathy and control group.

Protein–protein interaction (PPI) network analysis revealed that *Clu* exhibited the highest betweenness value (77.33), closeness (0.150442), and degree (10), ranking first among the three metrics. Consequently, it was identified as a hub gene in the network (Figure 4).

Further analysis of *Clu* expression across individual samples indicated a significantly higher expression in the SCM group than in the control group (*p* = 0.03; Figure 4).

### 3.5 WGCNA Results

**Figure 5:**
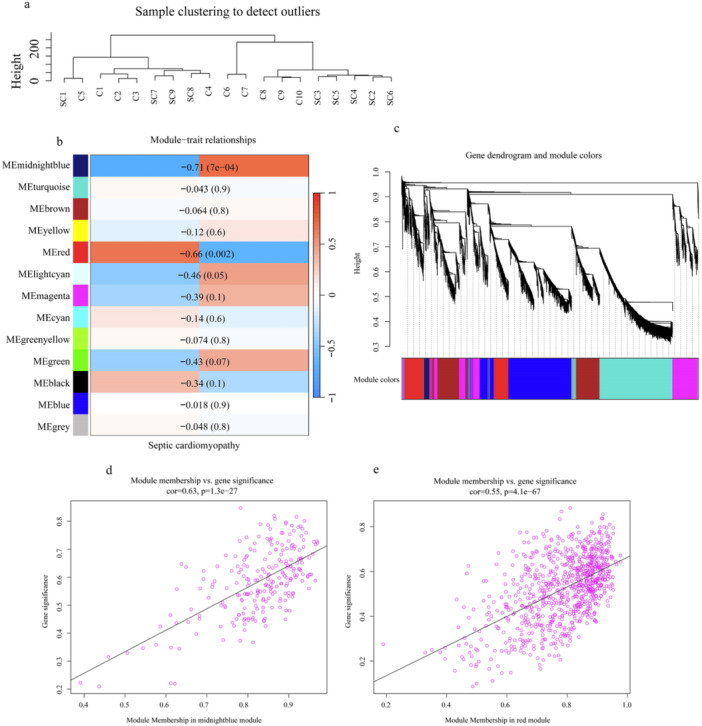
WGCNA Results. (a) Sample clustering diagram. (b) Correlation of 13 modules with septic cardiomyopathy phenotype. (c) Gene clustering map. (d, e) Pearson correlation analysis results of midnight blue and red modules with septic cardiomyopathy.

The WGCNA encompassed 12,874 genes, ultimately aggregating them into 13 distinct modules. Among these modules, the midnight blue and red modules exhibited a significant correlation with the SCM phenotype (*p* < 0.05). Consequently, these three modules were selected for the subsequent in‐depth analyses (Figure 5).

The Spearman rank correlation test revealed that both the midnight blue and red modules exhibited pronounced and significant correlations with the SCM phenotype. Subsequently, PPI networks were constructed for the genes within these two modules and a comprehensive network analysis was performed (Figure 5).

**Figure 6:**
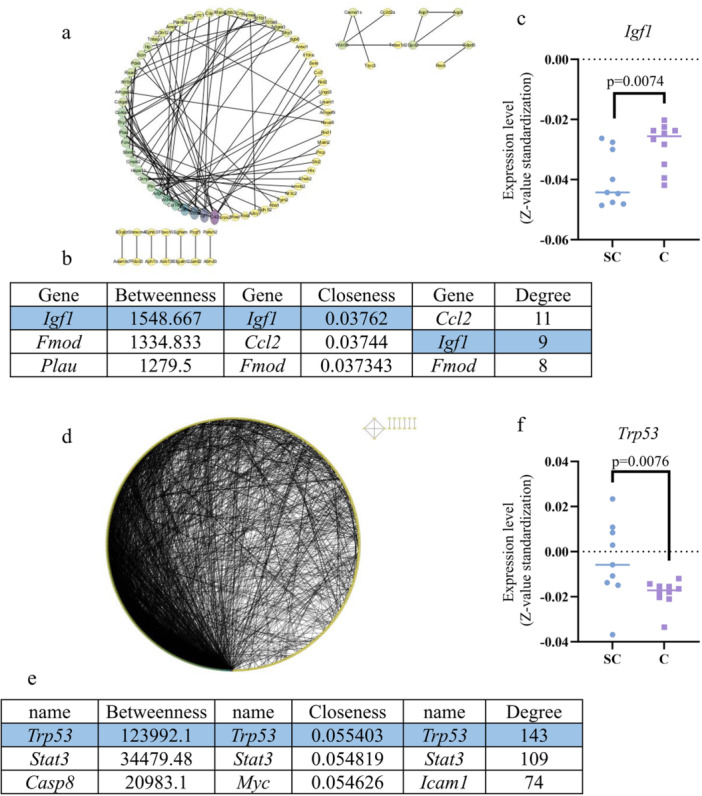
PPI analysis results of midnight blue and red modules. (a, b) PPI network graph and analysis results for midnight blue module. (c) Expression of Igf1 in septic cardiomyopathy and control groups. (d, e) PPI network graph and analysis results for red module. (d) the PPI network associated of the red module. (e) the analysis results highlighting key parameters related to the red module's PPI network. (f) Expression of *Trp53* in septic cardiomyopathy and control groups.

The analysis revealed that in the midnight blue module of the PPI network, *Igf1* emerged as the key gene, ranking first in terms of betweenness and closeness. Similarly, within the PPI network of the red module, *Trp53* was identified as a crucial gene, securing the top positions in betweenness, closeness, and degree based on the three algorithms (Figure 6).

Further analysis revealed that *Igf1* and *Trp53* expression was significantly lower and higher, respectively, in the SCM group than in the control group (*p* < 0.05; Figure 6).

### 3.6 GSEA and Immune Infiltration Analysis

**Figure 7:**
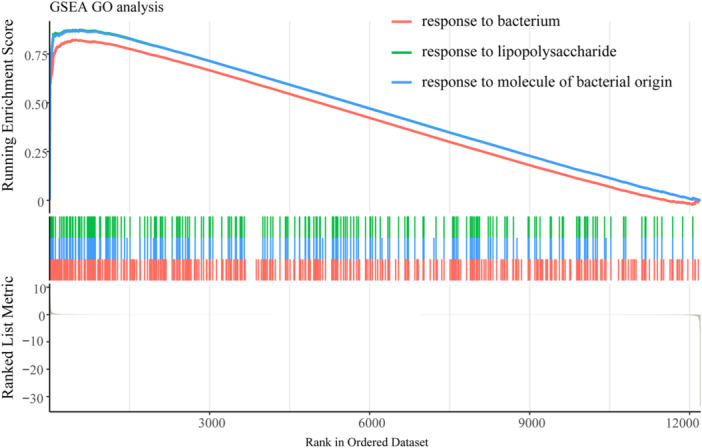
GSEA results. GSEA revealed significant enrichment of response to bacterium, response to lipopolysaccharide, and response to molecule of bacterial origin in SCM group compared to control group.

GSEA revealed a notable and statistically significant enrichment of gene sets associated with “response to bacterium,” “response to lipopolysaccharide,” and “response to molecule of bacterial origin” (Figure 7).

**Figure 8:**
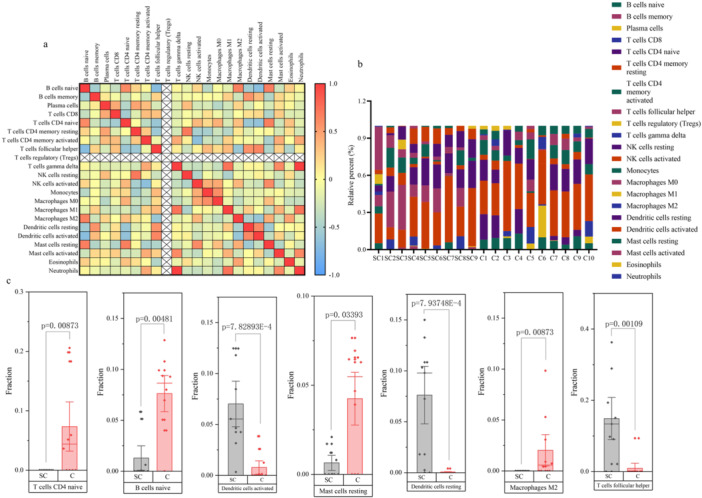
Immune infiltration analysis results. (a) Correlation analysis of different immune cell types. Displaying the correlation analysis results among different immune cell types. Red indicates strong correlation, while blue suggests weaker correlation. (b) Analysis results of immune cell infiltration in individual samples. Presenting the results of immune cell infiltration analysis across various samples. (c) Differential infiltration of immune cells between septic cardiomyopathy and control groups. Depicting the differential infiltration of immune cells between the septic cardiomyopathy and control groups.

Immune infiltration analysis revealed statistically significant decreases in the infiltration levels of T cells CD4 naive, B cells naïve, resting mast cells, and macrophages M2 in the SCM group than in the control group (*p* < 0.05), indicating a notable reduction in the number of these immune cell types (Figure 8).

Conversely, the infiltration levels of activated dendritic cells, resting dendritic cells, and follicular helper T cells statistically significantly increased in the SCM group (*p* < 0.05), indicating an enhanced presence of these immune cell subsets in SCM(Figure 8).

### 3.7 ceRNA and the Predictive Value of the Hub Genes

**Figure 9:**
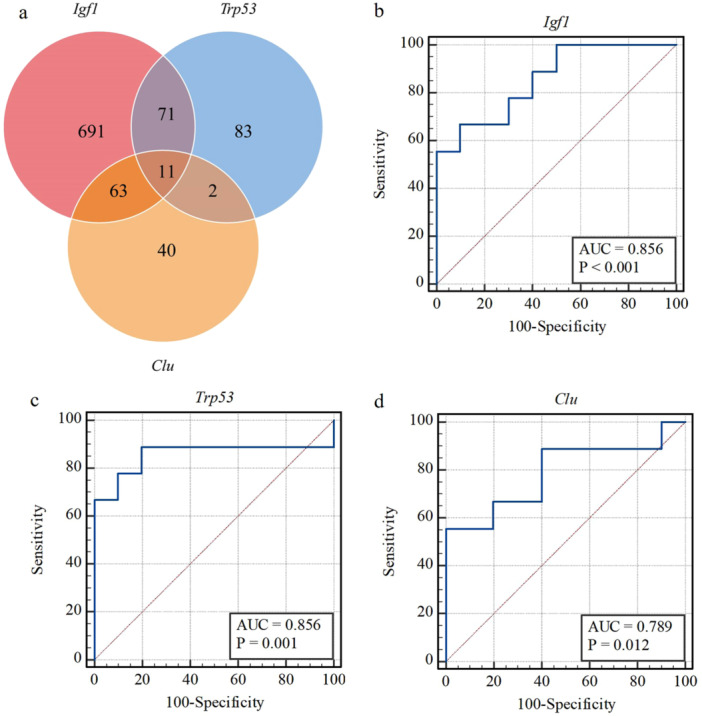
ceRNA regulation analysis and prediction results. (a) Venn diagram showed the regulation microRNA on hub genes. (b–d) ROC curve showed the prediction value of *Igf1, Trp53* and *Clu* on septic cardiomyopathy.

The miRNA prediction analysis indicated that 11 miRNAs concurrently regulated *Trp53, Igf1*, and *Clu*. The ROC curves demonstrated a strong correlation and predictive value for *Trp53*, *Igf1*, and *Clu* in the SCM (Figure 9). The areas under the curve (AUCs) for *Trp53*, *Igf1*, and *Clu* were 0.856, 0.856, and 0.789, respectively.

### 3.8 Mendelian Randomization Analysis

As no relevant SCM data were found in the IEU database, we used sepsis data as a substitute. The sepsis data in this study were numbered IEU‐b‐69 datasets from the IEU open GWAS database. The IGF1, CLU, and TRP53 data were numbered prot‐c‐2952_75_2, prot‐c‐4542_24_2, and prot‐a‐2238 datasets, respectively [19, 20].

Integrating the data on IGF1 and sepsis showed a significant causal association between IGF1 levels and sepsis incidence, and there were three associated SNPs. Genetic prediction showed that IGF1 levels negatively correlated with the risk of sepsis. The main effect estimate (β) was −0.31 (*p*‐value was 1.54E‐10).

The data analysis results for CLU and sepsis showed a significant causal association between CLU levels and sepsis incidence with two associated SNPs. Genetic prediction showed that the CLU level was positively correlated with the risk of sepsis. The main effect estimate (β) was 0.30 (*p*‐value was 9.16E‐06).

However, there was no significant causal association between TRP53 levels and incidence of sepsis. The main effect estimate (β) was 0.25 (*p*‐value was 0.18).

## 4 Discussion

Nowadays, SCM is being widely studied because of the high mortality, generally younger patients, and better baseline myocardial function [21]. Studies on SCM report a broad range of morbidity (10%–70%), which may be due to selection bias [4]. Rapidly progressive circulatory collapse generates patients who has more comorbidity die before admission to ICU. This may explain why the incidence of SCM was higher in the ICU. As the illness reverses and patients become relatively young, SCM cognition may save more families and lives in patients with sepsis and cardiac insufficiency. Diagnosis requires clear pathophysiological mechanisms, and effective therapies require early diagnosis and effective interventions. Unlike ischemic cardiomyopathy, SCM has a complex pathophysiology that has not yet been thoroughly elucidated. Therefore, there is currently no clinically confirmed method for treating reverse SCM.

In this study, we analyzed three different RNA sequencing methods to explore the mechanism of SCM with different intervening measures in a mouse model 24 h after model establishment. Nine samples from the SCM group and 10 samples from the control group were included. We identified 22 DEGs, 15 of which were upregulated and 7 were downregulated. Notably, *Mt1* was the most differentially upregulated gene, and *Actc1* displayed the most substantially downregulated gene.

*Mt1* is located on chromosome 16q13 in humans and contains at least 11 family member genes. They encode metallothioneins (MTs), which play essential roles in DNA damage and oxidative stress, except in metal homeostasis and against heavy metal toxicity [22]. In recent years, some studies have reported that MTs play key roles in many kinds of cardiomyopathy. Cong et al. found MT against age‐associated cardiovascular diseases mainly attributed to its inhibitory effect on NF‐κB [23]. Zhang et al. found that increased polymerization of copper‐responsive transition metal‐binding MT1/MT2 is consistent with damaged antioxidant defenses in diabetic cardiomyopathy [24].

Stroethoff et al. found. that ramelteon, a melatonin receptor agonist, acts on MT1 receptor induced cardio‐protection [25]. These suggest that upregulated *Mt1* aggravates the oxidative stress response in the SCM group. The *Actc1* gene encodes α‐actin, a highly conserved protein involved in various cell movements. It is related to myocardial contractility in dilated cardiomyopathy and hypertrophic cardiomyopathy [26, 27]. There are reasons to believe that left ventricular intrinsic contractility depression may be associated with *Actc1* downregulation in the SCM.

Thereafter, we used the PPI network for analysis using STRING 12.0. *CLU* was identified as the hub gene, and clusterin was the protein encoded by it. Clusterin is involved in multiple cellular processes, associated with cell cycle regulation, apoptosis and so on [28]. The current studies found that *Clu* can protect against myocardial injury in myocardial infarction [29, 30, 31]. Drugs associated with *Clu* may provide some evidences to improve the SCM. In patients with sepsis, plasma CLU levels are significantly reduced, with a greater decrease in non‐survivors than in survivors. This suggests that CLU is a protective molecule against circulating histones, linking its decline to worse outcomes, and highlighting its potential as a prognostic biomarker and therapeutic target [32]. Yagmur et al. found that clusterin levels were elevated in critically ill patients but lower in sepsis cases than in patients with non‐sepsis admitted to the ICU, inversely correlating with inflammatory biomarkers and unrelated to disease severity or mortality [33].

In addition to DEGs and PPI analyses, we used WGCNA to analyze the hub genes. We found that *Igf1* expression was significantly lower in the SCM group. Insulin‐like growth factor 1(IGF1), a peptide growth factor encoded by ‐*Igf1*, is a hormone that controls growth and metabolism and has anti‐apoptotic and pro‐survival capacity with an almost universal pattern of expression in most tissue [34]. Some studies have found that high expression of IGF1 is a core repertoire of reparative gene programs in fate‐mapped self‐renewing cardiac‐resident macrophages (RMs) [35]. IGF1 can increase the number of anti‐inflammatory macrophages in heart tissue through the induction of the M2 anti‐inflammatory phenotype macrophages [34]. Sun et al. found that the IGF1 inhibitor can promote apoptosis in acute myocardial infarction [36]. Although the pathogenesis of acute myocardial infarction and SCM is different, IGF1's protective effect is clear in cardiomyocytes. Previous studies had demonstrated that the GH/IGF‐1 axis was severely disrupted in patients with sepsis. IGF‐1 levels decrease significantly in patients with sepsis, correlating with disease severity. IGFBP‐3 is the primary determinant of IGF‐1 levels, reflecting disruption in the GH/IGF‐1 axis during sepsis [37].

Our results suggested that *‐Igf1* is a novel target for protecting cardiomyocytes from SCM. Moreover, *Trp53* was identified as a crucial gene in the PPI network of the red module and its expression was higher in the SCM group. *Trp53* is a gene expressed in mice and is called TP53 in humans. It encodes P53, a transcription factor that controls cell cycle initiation. It will start the apoptosis process in case of irreversible cell damage. Similar to other tumor suppressors, P53 plays a role in monitoring cell division. Increased TP53 may be associated with apoptosis in myocardial cells and cardiac insufficiency in SCM [38]. First, we first constructed an ROC curve to analyze the predictive value of *Trp53*, *Igf1*, and *Clu* in SCM. Second, *Clu* and *Igf1* demonstrated predictive values for SCM using Mendelian randomization analysis from the IEU database. This may provide new biomarkers to support clinical diagnosis. *Clu* and *Igf1* may converge on shared pathways in SCM, including oxidative stress, apoptosis, and immune regulation. *Clu* protects against oxidative damage, *Igf1* promotes cell survival, modulates immunity. These interactions highlighted their integrated roles in SCM pathogenesis.

We then probed the distinct gene expression patterns in the two groups using PCA. The main genes were *Fth1*, *Mt2* and *Saa3* in SCM group. *Saa3* is a potent pro‐inflammatory cytokine in various inflammatory diseases, especially in macrophages [39]. *Mt* seems to associate with *Fth1*, some studies found that MT inhibits ferroptosis via the SIRT6/NCOA4/FTH1 pathways [40]. While in the control group, *Actc1*, *Gsn*, *Hspb8*, and *Pln* are circled. GSN (Gelsolin) binds to actin monomers and filaments to prevent monomer exchange, and can combine with actin‐related transfer molecular [41]. *Pln*‐encoded proteins are key regulators of cardiac diastolic function. The expression of these genes may be associated with normal myocardial contractions [42].

We analyzed the results of the PCA and found that the differences in gene expression may be associated with whole cardiac tissue samples. In addition to myocardial cells, cardiac tissues contain fibrocytes and inflammatory cells. When SCM occurred, not only were the genes in myocardial cells related to the upregulated inflammatory response, but they were also accompanied by inflammatory cell infiltration and chemotaxis. As inflammatory cells exist, it is necessary to conduct an immune infiltration analysis.

Immune cell composition was estimated using the CIBERSORT algorithm. Although CIBERSORT was originally validated using human datasets, it has been successfully applied to murine transcriptomic profiles in several studies following gene orthology mapping and data normalization [43, 44].

Immune infiltration analysis was used to clarify the distribution and function of immune cells in the SCM. The reduction in naïve T/B cells, together with increased dendritic cells, likely indicates immune activation and antigen presentation in early sepsis, accompanied by partial immune exhaustion and cell recruitment to the cardiac tissue. The results showed low levels of T cells CD4 naive, B cells naïve, resting mast cells, macrophages M2 and, activated dendritic cells, resting dendritic cells, and follicular helper T cells in the SCM group. This may be related to the activation of anti‐inflammatory pathways. By integrating transcriptomic analysis with Mendelian randomization using the IEU OpenGWAS resource, our study introduces a genetics‐based layer of validation. The MR results for CLU and IGF1 provided causal support beyond simple associations, strengthening their relevance to SCM and highlighting them as promising targets for further mechanistic and therapeutic investigations.

### 4.1 Study Limitations and Future Research Direction

This study had several limitations. First, the study was predictive based on existing gene databases and lacked experimental confirmation. Second, there is a lack of clinical studies on hub genes, and we did not explore them as potential therapeutic targets for drug research. Additionally, the upstream and downstream regulation of hub genes was not analyzed. We aim to further address these limitations by conducting research at the cellular level, exploring the clinical aspects, and investigating hub genes as potential therapeutic targets. Furthermore, we aim to conduct a comprehensive analysis of the regulatory mechanisms both upstream and downstream of the hub genes.

## 5 Conclusion

SCM is sepsis‐induced cardiac dysfunction. *Clu* and *Igf1* have been identified as candidate genes in SCM and exhibit excellent predictive value. The onset of SCM is associated with the infiltration of various immune cells.

## Author Contributions

**Wei Liu:** conceptualization, formal analysis, methodology, software, writing – original draft. **Xi Zheng:** conceptualization, formal analysis, writing – original draft. **Dong Wang:** data curation, methodology, writing – original draft. **Jingyi Wang:** investigation, methodology. **Xincheng Li:** formal analysis, methodology, software. **Fei Li:** formal analysis, investigation. **Wenxiong Li:** conceptualization, formal analysis, supervision, writing – review and editing. **Jin Zhang:** Conceptualization, formal analysis, supervision, writing – review and editing.

## Funding

The authors received no specific funding for this work.

## Conflicts of Interest

The authors declare no conflicts of interest.
